# The effect of community groups and mobile phone messages on the prevention and control of diabetes in rural Bangladesh: study protocol for a three-arm cluster randomised controlled trial

**DOI:** 10.1186/s13063-016-1738-x

**Published:** 2016-12-19

**Authors:** Edward Fottrell, Hannah Jennings, Abdul Kuddus, Naveed Ahmed, Joanna Morrison, Kohenour Akter, Sanjit Kumar Shaha, Badrun Nahar, Tasmin Nahar, Hassan Haghparast-Bidgoli, A. K. Azad Khan, Anthony Costello, Kishwar Azad

**Affiliations:** 1 Institute for Global Health, University College London, London, UK; 2 Diabetic Association of Bangladesh, Dhaka, Bangladesh; 3 World Health Organisation, Geneva, Switzerland

**Keywords:** Diabetes, Intermediate hyperglycaemia, mHealth, Community mobilisation, Cluster randomised trial, Non-communicable disease

## Abstract

**Background:**

Increasing rates of type 2 diabetes mellitus place a substantial burden on health care services, communities, families and individuals living with the disease or at risk of developing it. Estimates of the combined prevalence of intermediate hyperglycaemia and diabetes in Bangladesh vary, and can be as high as 30% of the adult population. Despite such high prevalence, awareness and control of diabetes and its risk factors are limited. Prevention and control of diabetes and its complications demand increased awareness and action of individuals and communities, with positive influences on behaviours and lifestyle choices. In this study, we will test the effect of two different interventions on diabetes occurrence and its risk factors in rural Bangladesh.

**Methods/design:**

A three-arm cluster randomised controlled trial of mobile health (mHealth) and participatory community group interventions will be conducted in four rural upazillas in Faridpur District, Bangladesh. Ninety-six clusters (villages) will be randomised to receive either the mHealth intervention or the participatory community group intervention, or be assigned to the control arm. In the mHealth arm, enrolled individuals will receive twice-weekly voice messages sent to their mobile phone about prevention and control of diabetes. In the participatory community group arm, facilitators will initiate a series of monthly group meetings for men and women, progressing through a Participatory Learning and Action cycle whereby group members and communities identify, prioritise and tackle problems associated with diabetes and the risk of developing diabetes. Both interventions will run for 18 months. The primary outcomes of the combined prevalence of intermediate hyperglycaemia and diabetes and the cumulative 2-year incidence of diabetes among individuals identified as having intermediate hyperglycaemia at baseline will be evaluated through baseline and endline sample surveys of permanent residents aged 30 years or older in each of the study clusters. Data on blood glucose level, blood pressure, body mass index and hip-to-waist ratio will be gathered through physical measurements by trained fieldworkers. Demographic and socioeconomic data, as well as data on knowledge of diabetes, chronic disease risk factor prevalence and quality of life, will be gathered through interviews with sampled respondents.

**Discussion:**

This study will increase our understanding of diabetes and other non-communicable disease burdens and risk factors in rural Bangladesh. By documenting and evaluating the delivery, impact and cost-effectiveness of participatory community groups and mobile phone voice messaging, study findings will provide evidence on how population-level strategies of community mobilisation and mHealth can be implemented to prevent and control noncommunicable diseases and risk factors in this population.

**Trial registration:**

ISRCTN41083256. Registered on 30 Mar 2016 (Retrospectively Registered).

**Trial acronym:**

D-Magic: Diabetes Mellitus – Action through Groups or mobile Information for better Control.

## Background

### The global health importance of diabetes mellitus

Globally, type 2 diabetes mellitus (T2DM) and its complications are a growing concern among communities, health service providers and policymakers. The global prevalence of T2DM was estimated to be approximately 9% among adults in 2015, with around 75% of people living with T2DM in low- and middle-income countries [1]. Increasing rates of T2DM place a substantial burden on overstretched health care services, communities, families and individuals living with the disease or at risk of developing it. T2DM can lead to premature death; greatly increases the risk of cardiovascular disease; and, if not managed properly, can lead to serious morbidities, including nerve and renal complications [2]. Underlying the increasing prevalence of T2DM are complex genetic, environmental and lifestyle factors, including dietary changes and increases in risk factors such as smoking and lack of exercise.

### T2DM in Bangladesh

The Bangladesh Demographic and Health Survey (BDHS) of over 17,000 households across Bangladesh found that approximately 25% of people aged 35 years or older in rural areas had abnormal fasting glucose and approximately 11% were diabetic [3]. Analysing the data from the BDHS, Akter et al. [4] reported a higher prevalence of T2DM among older people but no significant differences between males and females. There was a significant difference between the poorest and richest households in diabetes prevalence (6.4% of the poorest households and 19.2% richest households); the difference was considerably lower for impaired fasting glucose levels (19.7% and 23.5%, respectively) [4]. Although the prevalence of diabetes was considerably higher in urban households (15.2%) than in rural households (8.3%), for impaired fasting glucose, the prevalence was slightly higher for rural households (23.5%) than for urban households (19%). Other studies have shown lower levels of dysglycaemia, with Bhowmik et al. [5, 6] reporting a combined 2009 prevalence of impaired fasting glucose and T2DM of around 12–13% among adults aged 20 years or older in the rural but urbanising Chandra District, close to Dhaka. Sayeed et al. [7] estimated the prevalence of impaired fasting glucose and T2DM to be approximately 17% in adults aged 20 years and older in rural Mymensingh District, although trend data suggest that the prevalence is now higher [5, 7, 8]. In a systematic review and meta-analysis of studies on cardiovascular diseases and T2DM in Bangladesh, Saquib et al. [9] estimated the prevalence of diabetes in the period 2006–2010 to be 9%, though their data suggest a lower prevalence for rural areas, and the overall prevalence described between 1995 and 2010 ranged from 2% to more than 21%, with a pooled prevalence of 6.7% [9]. The prevalence of impaired fasting glucose and impaired glucose tolerance were estimated to be 7% and 8.2%, respectively, when data from all studies were pooled. More recently, Islam et al. [10] estimated the prevalence of T2DM in rural Narail District to be 7.2% and impaired fasting glucose to be 5.3%. The scant representative epidemiological data and conflicting estimates of disease prevalence highlight a need for large-scale, community-based surveys in rural Bangladesh, where, at present, prevalence estimates of intermediate hyperglycaemia and T2DM range between 12% and 30%, with apparent regional differences.

Few studies have described the incidence of diabetes in South Asia, although Asghar et al. [8] estimated the incidence among adults older than 20 years of age in rural Bangladesh between 1999 and 2004 to be 16.4 per 1000 person-years, increasing to 49 per 1000 person-years when adjusted for the age and sex distributions of their sample. Their data show that the incidence among individuals with impaired fasting glucose is approximately four times higher than among the total population; thus, the incidence of T2DM among these individuals can be estimated to be as high as 20% per year.

Despite the high levels of T2DM and intermediate hyperglycaemia, awareness and control of the condition are limited. The BDHS survey found that 59% and 65% of affected women and men, respectively, were not aware of their elevated blood glucose levels and that only 37% of diabetic women and 31% of diabetic men were taking medication for their diabetes, with only 15% of women and 10% of men adequately controlling their blood glucose levels [3]. Reasons for low levels of awareness of one’s diabetic status and control of it include inadequate health services for diagnosing and treating diabetes effectively, not being able to afford regular health care and treatment, and communicable diseases remaining a priority for Bangladeshi health programmes [11]. Additionally, those who are less educated and of lower socio-economic status are significantly less likely to have their T2DM diagnosed and treated [11]. A study by Islam et al. [10] on knowledge of T2DM and glycaemic control among patients with T2DM in Dhaka found that 45.6% of patients in the study had good knowledge of diabetes, 37.7% had moderate knowledge of it and 16.7% had poor knowledge. Whereas the majority of respondents in the study identified changing lifestyle and diet as important to managing diabetes, specifics such as excessive sugar intake were not recognised as a risk factor, and the importance of exercise was identified as important by only 24.3% of respondents. In a separate study in rural Bangladesh, knowledge that diabetes can cause eye disease and can be controlled by regular exercise was found to be higher among men than among women [12]. Knowledge of diabetes prevention, control, consequences and risk factors has been found to be significantly associated with higher education, higher monthly income, family history of diabetes and a longer duration of a diagnosis of T2DM [10, 12].

### The need for further research and interventions

Research is needed to accurately describe the prevalence and patterns of T2DM, intermediate hyperglycaemia and risk factors for developing T2DM in rural Bangladesh. Although evidence demonstrates that lifestyle and other non-pharmacological interventions can prevent and delay the onset of T2DM [13], there is a lack of cost effective programs designed specifically for Bangladeshi populations, mindful of their own needs and resources.

### Mobile health

One such approach is mobile health (mHealth), broadly defined as the application of mobile technology to improve health and strengthen health systems. There is evidence that mHealth techniques can increase adherence to medication in those already diagnosed with a chronic disease [14]. There is also some evidence of mHealth influencing behaviour change. Authors of a review of 14 intervention studies (in the United States, Europe, Australasia and South Korea) specifically on phone messages used to decrease obesity found that 11 of the studies using short message service (SMS) messages had a statistically significant effect on weight loss, diet or exercise, though the interventions did differ substantially and questions remain about long-term impact [15]. Authors of a review of over 100 studies done over the last 25 years on chronic disease management and mHealth found that mobile messaging could assist with medicine adherence, although the results for chronic disease management were mixed, and more research into understanding and improving mHealth tools is needed [14].

Evidence of the effectiveness of mHealth interventions in low- and middle-income settings is mixed, with many lacking thorough evaluations [16, 17]. Of the mHealth interventions targeting T2DM in South Asia, a pilot study with patients with T2DM who received 1 year of SMS messaging regarding their treatment did reduce glycaemic levels as compared with the control group in India [18]. Ramachandran et al. [19] showed that the receipt of two to four mobile phone text messages per week with dietary and physical activity recommendations reduced the 2-year cumulative incidence of T2DM among professional men aged 35–55 years with impaired glucose tolerance by more than one-third in an individual randomised trial in southeastern India. Thus, although there is some evidence for mHealth having an impact on behaviour change and diabetic outcomes in a high-risk population, the effect at scale and in a general, rural population are unknown.

### Community mobilisation

There is good evidence for lifestyle interventions preventing and/or delaying the onset of T2DM [13, 20], and involving the community and peer support is a cost-effective means of promoting lifestyle changes. Community-based peer support as a method of T2DM control intervention is starting to be tested in low-income countries [20–24], and encouraging evidence is emerging. However, relatively small sample sizes, short intervention durations, focus on high-risk individuals and the lack of a counterfactual limits interpretation of cause and effect as well as potential impact at a general population level.

A randomised control trial is currently being conducted in Kerala, India [20]. Through group motivation sessions, diabetes prevention education sessions, handbook and participant workbooks, its aims are to decrease T2DM and to improve behavioural, psychosocial, clinical and biomedical measures in the community. The results of the 2-year intervention study are not yet available.

Community mobilisation through Participatory Learning and Action (PLA) is a specific approach to community interventions that fosters community engagement in the identification of problems and threats to their health, the design and implementation of solutions to tackle these problems, and reflecting on their success. This approach has been shown to substantially improve maternal and newborn health [25]. However, the PLA approach has not been tested on health issues beyond pregnancy, childbirth, neonatal [26, 27] and child health [28], and women’s and reproductive health [29].

## Methods/design

### Goals

The goals of the present study are to prevent intermediate hyperglycaemia^1^ and T2DM and to improve control of T2DM in Bangladesh.

### Objectives

The objectives of the present study are to design and implement community mobilisation and mHealth interventions for the prevention and control of diabetes in rural Bangladesh and to test their effect on intermediate hyperglycaemia and T2DM disease occurrence, management and risk factors in the population.

### Research questions and outcomes

Table 1 details all study research questions, outcome measures and definitions.

**Table 1:** Study outcomes and definitions

Outcome type	Outcome	Definition
Primary	The prevalence of intermediate hyperglycaemia and T2DM	Proportion of adults aged 30 years or older with WHO categorisations for intermediate hyperglycaemia (impaired fasting glucose or impaired glucose tolerance) and T2DM
	Two-year cumulative incidence of T2DM among individuals with intermediate hyperglycaemia at baseline	Proportion of adults aged 30 years or older with plasma glucose cut-off categorisations for intermediate hyperglycaemia at baseline who are categorised as T2DM at endline
Secondary	Blood pressure	Mean population diastolic and systolic blood pressure
	Prevalence of hypertension	Proportion of adults aged 30 years or older with systolic blood pressure ≥140 mmHg or diastolic blood pressure ≥90 mmHg or self-reported current treatment with anti-hypertensive medication
	Body mass index	Mean population BMI
	Prevalence of overweight and obesity	Proportion of adults aged 30 years or older with a BMI of 23 kg/m^2^ or more
	Abdominal obesity	Proportion of adult men and women aged 30 years or older with waist-to-hip circumference ratio >0.9 or >0.85, respectively
	Quality of life score	Mean health-related quality of life (EQ-5D)
	Psychological distress among self-reported diabetics	Mean SRQ score among adults aged 30 years and older with self-reported diabetes
Explanatory	Physical activity	Proportion of adults aged 30 years and older engaged in 30 minutes or more of physical activity per day on at least 5 days per week
	Intake of fruit and/or vegetables	Mean number of portions of fruit or vegetables consumed per adult aged 30 years or older per day
	Population knowledge about diabetes risk factors, symptoms and complications	Proportion of adults aged 30 years and above who are (a) able to name at least one cause of diabetes, (b) able to report at least one symptom of diabetes, (c) able to report at least one complication of diabetes, (d) able to recognise complications of diabetes when prompted, (e) able to report at least one way to reduce the risk of getting diabetes and (f) able to report at least one way to control diabetes if diagnosed
	Self-awareness of diabetic status	Proportion of diabetics who correctly report their diabetic status
	Receipt of treatment or advice for diabetes	Proportion of diabetics receiving care or advice from a medical professional

*Abbreviations: BMI* Body mass index, *SRQ* Self-Reporting Questionnaire, *T2DM* Type 2 diabetes mellitus, *WHO* World Health Organisation

#### Primary research questions

The following are the primary research questions of the study:

1. What is the effect of a participatory community mobilisation intervention on a) the prevalence of intermediate hyperglycaemia and T2DM and b) two year cumulative incidence of T2DM among individuals with intermediate hyperglycaemia at baseline?
2. What is the effect of an mHealth health promotion and awareness intervention on a) the prevalence of intermediate hyperglycaemia and T2DM and b) two year cumulative incidence of T2DM among individuals with intermediate hyperglycaemia at baseline?

#### Ancillary questions

The following are ancillary questions of the study:

- What is the effect of a) a participatory community mobilisation intervention and b) an mHealth health promotion and awareness intervention on population:
-
  - Blood pressure
  - Prevalence of hypertension
  - Body mass index
  - Prevalence of overweight and obesity
  - Prevalence of abdominal obesity
  - Health-related quality of life
  - Psychological distress among self-reported diabetics

#### Explanatory outcomes

In addition to the questions above, which will be answered in terms of primary and secondary outcomes (Table 1), five additional explanatory outcomes will be measured to better understand intervention processes and mechanisms of action in terms of changes in the following:

1. Population physical activity levels
2. Population intake of fruit and vegetables
3. Population knowledge about diabetes risk factors, symptoms and complications
4. Self-awareness of diabetic status
5. Proportion of diabetics receiving treatment or advice for diabetes

### Trial design overview

The mHealth and community mobilisation interventions will be evaluated using a three-arm cluster randomised controlled trial design. The rationale is that each intervention is applied to the entire community and is not based on individual enrolment. Ninety-six villages will be randomised to either the mHealth intervention, community mobilisation or the control arm, and, given the nature of the interventions, allocation is not masked. Baseline and endline sample surveys measuring the prevalence of intermediate hyperglycaemia, diabetes, hypertension and various chronic disease risk factors, quality of life, as well as knowledge and care-seeking practices will be conducted before and after intervention delivery, respectively. Individuals identified at baseline as having intermediate hyperglycaemia will be followed after the interventions to determine the incidence of T2DM among this high-risk population.

### Setting

**Fig. 1:**
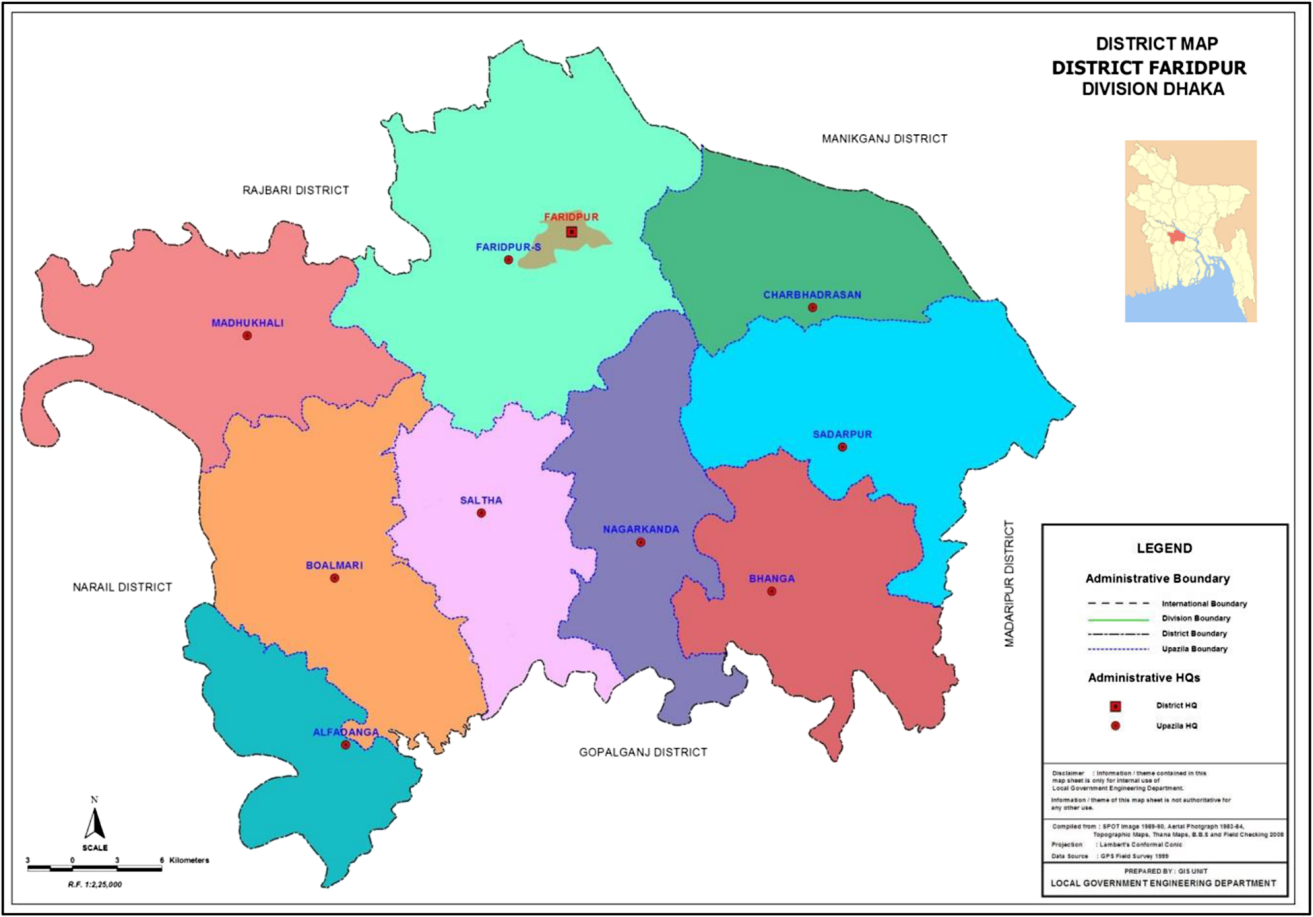
Map of Faridpur District divided into nine upazillas (source: Local Government Engineering Department, Bangladesh)

Faridpur District has a population of over 1.7 million people in an area of just over 2000 km^2^ and is situated on the banks of the Padma River (Fig. 1). The district has a mainly agricultural economy, with the main crops being jute and rice. Large rivers throughout the district are prone to flooding, which can hinder travel and health care access in the area. The population is mainly Bengali, as is the case in most of Bangladesh, and almost 90% of the population of Faridpur are Muslim, with the remaining population largely Hindu. Administratively, Faridpur District is divided into nine upazillas, which in turn are divided into unions of approximately 25,000 population per union. The number of unions per upazilla in Faridpur District varies from 4 to 17. Each union is comprised of a number of mauzas, traditional administrative units that can be conceptualised as a village or collection of villages. Four upazillas in Faridpur District have been purposefully selected on the basis of being accessible to the district headquarters of the Diabetic Association of Bangladesh (BADAS) in Faridpur Sadar: These are Boalmari, Saltha, Madhukhali and Nagarkanda. For each of these upazillas, the 2011 Bangladesh Census [30] was used to identify all mauzas with at least one village of between 750 and 2500 population size, 96 of which were selected for inclusion in the study.

In Bangladesh, primary care is provided by the government at union health and family welfare centres and at community clinics. In- and outpatient services are provided at subdistrict (upazilla) health complexes and hospitals, and tertiary care is provided at district hospitals and medical college hospitals. Inadequate facilities and trained health care providers, short supplies of medicines, and low responsiveness of services are challenges faced by the health care system in Faridpur District. Private health care provision is also available in Faridpur, including at the Diabetic Association Medical College Hospital in Faridpur Sadar.

The trial is implemented by the BADAS Perinatal Care Project (PCP), a non-profit, voluntary socio-medical organisation registered with the Ministry of Social Welfare that works closely with the Government of Bangladesh in collaboration with the Institute for Global Health, University College London.

### Study population

In intervention areas, the target group for the mHealth and community mobilisation interventions are male and female permanent residents aged 30 years or older because this is the group that we expect to benefit the most from the community-level interventions. Note, however, that both interventions will be available and accessible to any community member, including health care providers. People are considered permanent residents of a village if the house that they normally live in is in that village. Temporary residents whose permanent home is in a village not included in the study or included in a different arm are not considered part of the intervention target population.

### Interventions

#### mHealth

The mHealth intervention will consist of twice-weekly health behaviour-related and awareness-raising voice messages sent to individuals’ mobile phones over a period of 18 months. Message content will be based on findings from formative research in the study area, application of behaviour change theories, review by medical experts and project staff, and specification of a creative brief which will be used by scriptwriters to create messages in a range of formats, such as mini-dramas, dialogues and songs. These will be performed by actors and recorded, and a signature jingle will be added to make the messages recognisable as coming from BADAS-PCP. Each message will be recorded in Bangla in Dhaka, and recipients will receive distinct messages on two different occasions each week during the 18-month intervention period. Messages will be sent to all subscribed mobile phones using a service provided by MWorld (Dhaka, Bangladesh), a commercial third party.

The intervention will be available to all individuals with access to a mobile phone in the study areas. Individuals will need to register for the messaging service by providing their mobile phone number to community recruiters. Recruiters will coordinate community engagement activities, which will include community meetings, and mass communication methods, such as posters, leaflets, loudspeaker broadcasting and presentations at public service points (e.g., markets, clinics, places of worship). In addition to these recruitment drives, individuals will be able to self-enrol at any time throughout the study period.

A behaviour change theory-based approach to the mHealth intervention is likely to increase its chances of working [19, 31]. As such, the intervention will be developed using the capability, opportunity, motivation and behaviour (COM-B) theory for understanding behaviour [32] and the Theoretical Domains Framework (TDF) to encourage change [33, 34].

The COM-B framework focuses on the interaction between capability, opportunity and motivation to generate behaviour. *Capability* is defined as an individual’s psychological and physical capacity to engage in the activity concerned. It includes having the necessary knowledge and skills. *Motivation* is defined as all the brain processes that energise and direct behaviour, not just goals and conscious decision-making. It includes habitual processes, emotional responding and analytical decision-making. *Opportunity* is defined as all the factors that lie outside the individual that make the behaviour possible or prompt it. The different elements are inter-related. For example, opportunity can influence motivation, as can capability; engaging in a behaviour can alter capability, motivation and opportunity. The TDF framework (which corresponds with COM-B) was developed through consensus by behaviour change specialists and has been validated and refined [33, 35]. The framework simplifies and integrates existing behaviour change theories to make them accessible. It identifies 14 overarching behavioural domains that can be mapped according to capability, opportunity and motivation [35]. By categorising behaviour, different behaviour change techniques can be drawn on in order to encourage behaviour change [36]. Behavioural domains in the formative research that correspond with specific barriers to and enablers of behaviour change (e.g., knowledge, environmental context, beliefs about consequences) will be identified. In order to systematically address enablers and barriers, behaviour change techniques, such as shaping knowledge, modelling behaviour, and information about consequences, will be applied to the messages. Application of COM-B and the TDF to formative research and engagement with community members will involve an element of ‘behavioural diagnosis’ whereby it will be possible to identify areas of leverage for behaviour change within our target population.

Whilst the precise details of the mHealth intervention will emerge from the formative work, some general principles and descriptors of the intervention using the World Health Organisation (WHO) mHealth Technical and Evidence Review Group standard taxonomy of mHealth activities can be specified [37]. The target will be health promotion and disease prevention for the avoidance and continuous control of non-communicable disease risk factors, particularly those associated with T2DM, among the adult population using mobile phone voice messages as a communication channel.

#### Community mobilisation

The community mobilisation intervention will be an adaptation of a participatory women’s group intervention implemented and evaluated in several settings and shown to be effective at reducing neonatal mortality [27, 25]. The intervention will entail the initiation and facilitation of separate male and female participatory groups comprising approximately 20 members each. Groups will be open to all community members of any age; however, people with T2DM and high-risk individuals will be particularly encouraged to attend. Separate groups will be established for men and women because this is the approach most likely to achieve true participation within the rural Bangladeshi context, and there will be an equal number of male and female groups within each community mobilisation intervention cluster, with a minimum of one male and one female group per cluster. Joint meetings of men’s and women’s groups, as well as the wider community, will be encouraged. Enough groups will be established to ensure population coverage of approximately 1 group per 600 population or 1 group per 200 adults; however, the requirement of at least one male and one female group per cluster may increase population coverage beyond this target.

**Fig. 2:**
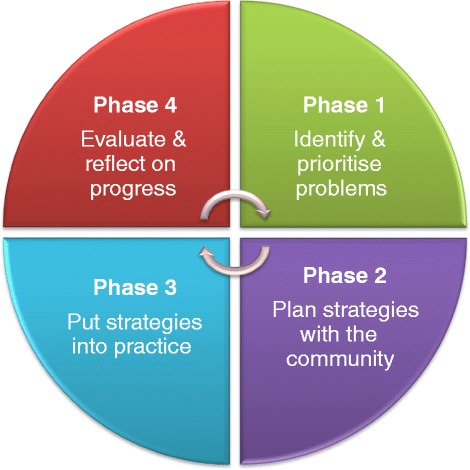
Four-phase participatory learning and action cycle underpinning the community mobilisation intervention

The groups will proceed through a series of 18 monthly meetings and several wider community-level meetings following the four phases of PLA (Fig. 2). Phase 1 will be focused on problem identification, whereby participants themselves identify and prioritise factors that affect their health, specifically threats that increase their risk of developing or failing to manage diabetes. Phase 2 involves the collective design of strategies that participants and their communities can implement to address the problems and threats identified in phase 1. During phase 3, the participants and community implement these strategies. In phase 4, the participants reflect on and evaluate the success of the strategies they have implemented. Problems that might be identified in phase 1 may include lack of awareness of signs and symptoms of diabetes, limited opportunity for physical exercise in a culturally appropriate or secure environment, or lack of choice in locally available food types. The community-led strategies that might be implemented may include large community meetings to raise awareness, community-led exercise groups, or cookery workshops highlighting healthier food types and cooking methods. Each meeting will comprise a range of activities using picture card games, discussion, role-playing, and storytelling to raise awareness and improve support, treatment and preventive behaviours for the population as a whole and for individuals with T2DM and their families. Those who attend groups will be encouraged to share their learning and key messages with other members of the community who were unable to attend, such as through household visits and establishing links with other groups in the area.

Participatory groups will be led by salaried group facilitators, each required to coordinate and facilitate several participatory group meetings per month. All facilitators will have a minimum of higher secondary school education, will be recruited from the study areas, and will receive 1 week’s training on group facilitation and basic health messages related to chronic disease prevention and control, with a particular focus on T2DM. The facilitators will also be provided with a community facilitation manual with essential information on T2DM as well as its causes and control. The community facilitation manual will be developed during the formative phase of the project and will be aligned with national and international standards and based on materials developed by BADAS. Facilitators will be equipped with simple picture cards and flip charts graphically conveying health messages to stimulate discussion and critical thinking. Male groups will be led by a male facilitator and female groups will be led by a female facilitator. Facilitators will be motivated and provided with information and support through mobile phone messages and by support visits by two female participatory group coordinators based in Faridpur and a community group manager based in Dhaka.

#### Health system strengthening

All study clusters (intervention and control) will receive health system-strengthening activities. The reason to include a health system-strengthening component in our study is twofold. First, there is the ethical imperative to ensure that the control areas also benefit from the study. Second, we expect some degree of a functioning ‘supply side’ local health system to be necessary for the success of the ‘demand side’ mHealth and community mobilisation interventions. Specific health system-strengthening initiatives will be tailored according to the situation analysis conducted during the formative phase of the project but are likely to include the training/refresher training of formal and informal health care workers in the prevention, diagnosis and treatment of T2DM, and the development of essential equipment inventories.

### Randomisation

Using available maps, 24 mauzas from each of the 4 upazillas were purposefully selected on the basis of appearing to avoid contiguity between mauzas. A single eligible village within the selected mauza was designated the study cluster. In this way, it was intended that all study villages (clusters) would be separated from one another by a buffer zone of mauzas and villages not included in the study.

**Fig. 3:**
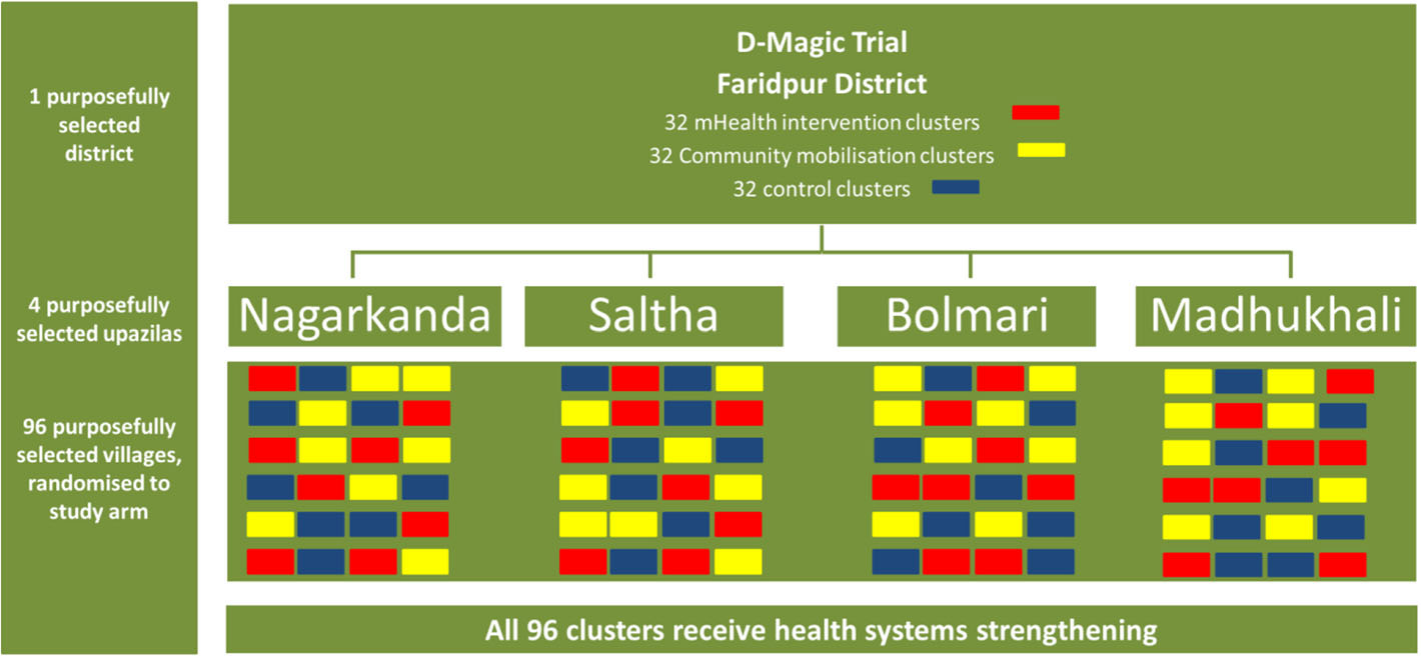
D-Magic cluster randomised trial design

By stratified randomisation, the 96 villages will be randomly allocated to either the mHealth intervention arm, the community mobilisation arm or the control arm (32 in each), with each upazilla constituting 1 stratum (Fig. 3). The name of each village will be written on pieces of paper, colour-coded by upazilla, which, when folded, will be indistinguishable from each other. For each upazilla, the 24 folded pieces of paper will be placed into a bottle and then drawn by community leaders and representatives at a public community orientation meeting in Faridpur attended by the project director, project manager and independent observers. The first eight villages drawn from the bottle will be allocated to ‘arm A’, the next eight villages to ‘arm B’ and the final eight villages to ‘arm C’. After all 96 villages have been allocated to an arm, each of the 3 arms will be randomly assigned to either the mHealth intervention, community group intervention or control group by simultaneously drawing arm letter and intervention allocation from 2 separate bottles. The entire randomisation process will be filmed, and photographs will be taken to document every stage.

### Minimising contamination

Every effort was made using available maps and population data to purposefully select study villages (clusters) to be non-contiguous to minimise contamination between intervention clusters and between intervention and control clusters. Further, exclusion of temporary or migratory individuals from the study surveys will minimise contamination between clusters.

### Sample size

Sample size was determined using equations of Hayes and Bennett [38] and Hayes and Moulton [39]. The trial, with 3 treatment arms, will include 32 mHealth intervention clusters, 32 community mobilisation intervention clusters and 32 control clusters, with an average population of 487 adults aged 30 years or older per cluster. As detailed in the Background section above, estimates of intermediate hyperglycaemia and T2DM prevalence in Bangladesh vary [3–8]; therefore, our sample size calculation assumes a mid-range baseline/control prevalence of 17%. In the absence of published values, using an estimated, moderate between-cluster coefficient of variation (*k*) of 0.265, a sample of 130 adults aged over 30 years per cluster will give 80% power at 95% confidence to detect a minimum of 21.5% reduction in disease prevalence in intervention clusters relative to control clusters.

Assuming a baseline prevalence of abnormal fasting glucose of 11%, the baseline surveys of 130 adults per cluster will identify approximately 15 individuals with intermediate hyperglycaemia per cluster. With an assumed 25% two-year cumulative incidence of T2DM among these high-risk individuals, two-year follow-up will give our study 78% power at 5% significance to detect a one-third (33%) reduction in cumulative incidence in intervention clusters relative to control clusters using a between-cluster coefficient of variation of 0.265. An additional 10% will be added to the sample list to allow for refusals, inability to contact and loss to follow-up, bringing the total target sample size to 143 per cluster, or 13,728.

### Impact evaluation

Our trial has two primary outcomes. First is the combined prevalence of intermediate hyperglycaemia (i.e., impaired fasting glucose or impaired glucose tolerance) and T2DM among adults aged 30 years or older. We will use WHO and International Diabetes Federation definitions and blood glucose cut-offs for normoglycaemia, impaired fasting glucose, impaired glucose tolerance and T2DM, as summarised in Table 2 [40]. These levels have been used in previous studies in Bangladesh and in other Asian countries [3, 4].

**Table 2:** Glycaemia definitions and diagnostic criteria to be used in the D-Magic trial

Definition		Diagnostic criteria
Normoglycaemia		Fasting plasma glucose <6.1 mmol/L
Intermediate hyperglycaemia (sometimes termed *pre-diabetes*)	Impaired fasting glucose	Fasting plasma glucose ≥6.1 mmol/L to <7.0 mmol/L *and* Two-hour post-ingestion of 75-g glucose load plasma glucose <7.8 mmol/L
	Impaired glucose tolerance	Fasting plasma glucose <7.0 mmol/L *and* Two-hour post-ingestion of 75-g glucose load plasma glucose ≥7.8 mmol/L to <11.1 mmol/L
Type 2 diabetes mellitus		Fasting plasma glucose ≥7.0 mmol/L *or* ^a^ Two-hour post-ingestion of 75-g glucose load plasma glucose ≥11.1 mmol/L

Adapted from [40]

^a^Diabetes cannot be excluded without 2-h post-oral glucose load test

Our second primary outcome is the cumulative 2-year incidence of T2DM among individuals identified with intermediate hyperglycaemia during our baseline survey.

### Data collection and management

#### Sampling frame and sample

A sampling frame will be developed specifically for this study by listing all households and their eligible members (non-pregnant permanent residents aged 30 years or older) within each of the study clusters. A sample of 143 adults aged 30 years or older will be randomly selected from this sampling frame from each study cluster using multi-stage simple random sampling. In the first stage, 143 households with at least 1 eligible adult resident will be selected using probability proportional to size sampling. At the next stage, a single eligible adult will be selected for inclusion in the survey using simple random sampling.

#### Surveys

Sampled individuals will be visited in their household and informed of the study, and their verbal consent will be obtained. Sampled individuals and data collectors are unlikely to be aware of their village’s allocation to the intervention or control arm at the time of the baseline survey, but they are likely to be aware of allocation at the endline survey owing to the unblended nature of allocation. All sampled individuals in a single cluster will be informed of the anthropometric, blood glucose and blood pressure measurement requirements of the study and will be requested to attend a local centre on the morning of a specified day following an overnight fast. The centre will be established by the field team for the purposes of the study and will be at a central, convenient location in the village. Collection of questionnaire data will take place at the respondents’ homes before or after the physical measurements or at the time of physical measurement in the testing centre.

Following extensive training and field practice, pairs of trained fieldworkers (one male and one female) with at least higher secondary school qualifications will be trained to measure blood pressure, blood glucose concentration, body weight, height, and waist and hip girth using standard methods. Blood pressure will be measured using the OMRON HBP 1100 Professional Blood Pressure Monitor (OMRON Healthcare, Kyoto, Japan). Two sitting measurements will be taken at approximately 5-minute intervals, and the respondent’s blood pressure will be obtained by averaging these measurements. Measurements of height, weight, and waist and hip girth will be taken with subjects wearing light clothes and without shoes. The weighing tools will be calibrated daily by known weight. For height, the subject will stand in erect posture vertically touching the occiput, back, hip and heels to the wall while gazing horizontally in front and keeping the tragus and lateral orbital margin in the same horizontal plane. Waist girth will be measured by placing a non-stretchable plastic tape horizontally midway between the 12th rib and the iliac crest on the mid-axillary line. Similarly, hip circumference will be measured by taking the extreme end posteriorly and the symphysis pubis anteriorly. Physical measurements will be recorded on specifically designed forms and later entered into a mobile phone data entry system.

Blood glucose will be measured using the OneTouch Ultra glucometer (LifeScan, Inc., Milpitas, CA, USA) in whole blood obtained by finger prick from capillaries in the middle or ring finger after an overnight fast of 8–12 h, an approach that is widely used in resource-limited countries [4]. The OneTouch Ultra glucometer automatically converts whole-blood glucose readings to the equivalent plasma value, so no additional conversion will be required. All individuals will then receive a 75-g glucose load dissolved in 250 ml of water and will have a repeat capillary blood test within 5 minutes of 120 minutes post-ingestion to determine glucose tolerance status and differentiate between individuals with intermediate hyperglycaemia and those with diabetes according to WHO criteria (Table 2).

Detailed information on the socio-demographic characteristics of all sampled individuals will be collected by the trained fieldworkers using a structured survey instrument adapted from the WHO STEPwise tool [41] and the 2011 Bangladesh Demographic and Health Survey [3]. The instrument will be designed to measure the background demographic and socio-economic characteristics, lifestyle and behavioural risk factors, diabetes awareness indicators, health-seeking behaviour, and costs of care-seeking among study participants. The EQ-5D questionnaire will be used to measure quality of life [42]. Questionnaire data will be gathered using Samsung Galaxy Grand Prime (Samsung, Seoul, South Korea) large-screen smartphones (US$200) using ODK Collect open-source software.

Fieldworker activity will be monitored by a team of supervisors with experience in field data collection and survey techniques. Supervisors will observe data collection processes and conduct some repeat measurements for verification in a random selection of cases. One supervisor will have the sole responsibility of collecting data from each individual fieldworker’s mobile phone onto a laptop every 3 days (maximum) and transferring these data to the district project office for data-checking before final transfer of the data to Dhaka. Individuals unable to participate in the data collection at the testing centre or on the specified day will be followed and asked to participate in the study at a later date.

#### Implementation research and process evaluation

Detailed quantitative and qualitative process evaluation research will be done in intervention and control areas throughout the trial to consider the effect of context on interventions, to describe the implementation of the interventions, and to help explain any observed intervention effect or lack of effect. Process data will be used throughout implementation to inform intervention design and delivery and will help to develop theory about how the interventions impact health outcomes. This will enable assessment of the feasibility, acceptability, scalability and external validity (or replicability) of the interventions.

### Analysis

#### Interim analysis and stopping rules

A meeting of an independent Data and Safety Monitoring Board (DSMB) will be convened according to the DAMOCLES charter after entry and cleaning of baseline survey data and approximately 9 months’ worth of implementation and process data. The DSMB will be tasked with providing an independent, objective review of the study implementation and baseline data and advise on any extension or modification of the trial. In particular, it will review the following:

1. Process indicators, including community group coverage, mHealth coverage (subscription rates) and adherence to the implementation plan
2. The adequacy of the sample size and the validity of sample size calculation assumptions about baseline prevalence and incidence and intra-cluster correlation coefficients
3. The comparability of treatment arms according to baseline survey data
4. Data quality
5. Endline survey design
6. The proposed trial analysis plan, including methods for adjustment of stratification and clustering and intervention effect measures to be reported

Intervention allocation will be masked to the DSMB. There are no stopping rules, because we do not expect the intervention to have adverse effects at either the cluster or participant level.

#### Intention-to-treat analysis

The trial will test the effect of a community mobilisation intervention and an mHealth intervention, as described previously, relative to a control group. We do not intend to directly compare the effects of each intervention relative to the other. Analysis will be based on intention to treat at the individual and cluster levels as appropriate for the outcome measures. The intention-to-treat population includes only adults aged 30 years or older who are permanently residing in the village in which they are surveyed. Participants with missing data on the primary outcomes will be excluded from primary outcome analysis but may be included in secondary outcome analysis, depending on data completeness. All analyses will be carried out with adjustment for the stratified, clustered study design, without and with adjustment for potential confounders resulting from imbalance between study clusters, which will be selected on the basis of their importance as a determinant of the outcomes rather than the size or significance of any difference between arms. Estimates of the intervention effects will be presented with 95% confidence intervals. No correction for multiplicity will be made. Analysis will be conducted using STATA statistical software (StataCorp, College Station, TX, USA).

#### Economic evaluation and equity impact analysis

Cost and cost-effectiveness analyses will take a societal perspective, assessing the economic impact for all parties affected by the interventions, including implementing agencies (project or program costs), health care providers (at local and national levels), and users or households. Program costs will be collected prospectively from the project accounts, and data on provider costs will be collected retrospectively from a random sample of primary care providers in the project area. Costs incurred by users or households will be collected during household and other follow-up surveys. All costs will be estimated from both a financial and an economic perspective, adjusted for inflation using the Bangladesh Consumer Price Index (CPI) and presented in international dollars. Incremental cost-effectiveness ratios will be evaluated in terms of the cost per case of intermediate hyperglycaemia and T2DM prevented, cost per case of diabetes prevented among individuals with intermediate hyperglycaemia at baseline, cost per millimetre of mercury reduction in systolic blood pressure, and other secondary outcomes to be detailed in a separate economic evaluation protocol to be submitted for publication. The robustness of the cost-effectiveness results will be assessed through a series of sensitivity analyses. In addition to cost-effectiveness analysis, an equity-impact analysis will be conducted to assess whether the interventions have improved the equity of health service delivery and are improving the health status of those most in need.

### Timetable

**Fig. 4:**
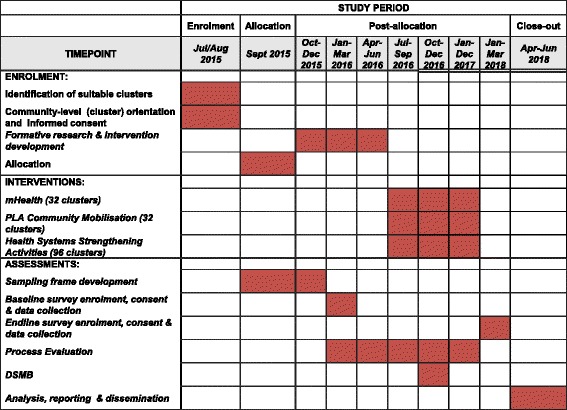
Study time frame. *DSMB* Data and Safety Monitoring Board, *PLA* Participatory Learning and Action

The intervention period is between June 2016 and December 2017. A time schedule for project activities is displayed in Fig. 4.

### Ethics

#### Approvals

The trial has been reviewed and approved by the University College London Research Ethics Committee (4766/002) and by the BADAS Ethical Review Committee (BADAS-ERC/EC/t5100246). The trial has been registered and assigned an International Standard Randomised Controlled Trial number (ISRCTN41083256).

#### Community consultation

Community leaders will be identified and approached to obtain permission to deliver the interventions and conduct the evaluation surveys. Orientation meetings will be held to explain the project aims, and community leaders will be invited to observe the randomisation process. The concept of random sampling for inclusion in the study surveys will be explained. Further, community advisory committees composed of community representatives and leaders will be established in each study upazilla with the purpose of advising and informing the research process and linking the research team to the community [43, 44].

#### Consent

Individual participation in the community meetings and receipt of the mHealth messages will be on a voluntary basis, and participants can choose to withdraw from either intervention at any time. Prior to all interviews and focus group discussions, the purpose of the data collection will be explained, an information sheet will be provided, and consent from the interviewee will be obtained. Respondents will be told that they can decline to participate in the study and can refuse to answer any question. Access to the identifiable individual-level data will be restricted to surveillance and data entry staff as well as analysts from the study team.

#### Treatment of illness in study areas

Fieldworkers will be trained to refer any individuals identified with possible intermediate hyperglycaemia, diabetes or hypertension to an appropriate health care facility for consultation and confirmatory testing.

#### Benefits to control areas

We have equipoise on the interventions under test: the effect of an mHealth messaging intervention or participatory groups on T2DM and its risk factors is unknown in the Bangladeshi context. It is therefore important to test the effect of this intervention using a randomised trial design. It is ethically important that the control areas also benefit from the study. They will do so in two ways. First, they will benefit directly through the health system-strengthening activities that will be undertaken in both study arms. Second, they will benefit indirectly through the strengthening of the evidence base on the burden of T2DM and its risk factors and our advocacy activities at the local, national and international levels.

#### Scalability

The potential for scaling up the interventions under investigation in Bangladesh is high. Scale-up would be facilitated by the fact that the project partner (BADAS) is the largest health care provider in Bangladesh after the government and has strong links with government agencies as well as private sector agents relevant to scale-up, such as mobile telecommunications companies.

#### Role of funder

Peer review of the grant application influenced the design of the study and study outcomes. Beyond that, the funder will have no role in the design of the study; the data collection, analysis and interpretation; or the write-up of the findings.

### Dissemination

Successful completion of the Bangladesh D-Magic trial will contribute to the science of the evaluation of complex community-based interventions, behaviour change and implementation research. Beyond these academic endeavours, we anticipate that our study activities will benefit individuals and communities in our study areas, service providers and policymakers in Bangladesh, and advocacy and implementation groups tackling diabetes in Bangladesh and internationally. Specifically, the data generated from the epidemiological description of the burden of diabetes and its risk factors in Bangladesh, as well as the anthropological perspectives added through formative research, will deepen understanding of the issue within the study context. Impact and process evaluation of two discrete interventions will help to identify cost-effective ways to address the burden of diabetes and non-communicable disease risk factors in a low-income, rural South Asian setting. Study activities and findings will be disseminated to a range of audiences, including study participants, service providers and policymakers, through a range of appropriate means, including workshops, dissemination meetings and peer-reviewed publications.

## Discussion

This protocol describes the Bangladesh D-Magic trial which, through implementation science methods, mixedmethods process evaluation and rigorous impact evaluation using a three arm cluster randomised controlled design, will increase our understanding of diabetes and other non-communicable disease burdens and risk factors in rural Bangladesh. Study findings will provide evidence on how population-level strategies of community mobilisation and mHealth can be implemented to prevent and control non-communicable diseases and risk factors in this population.

### Trial status

The trial is ongoing.

## Abbreviations

BADAS: Diabetic Association of Bangladesh
BDHS: Bangladesh Demographic and Health Survey
BMI: Body mass index
COM-B: Capability, opportunity, motivation and behaviour
CPI: Consumer Price Index
DSMB: Data and Safety Monitoring Board
mHealth: Mobile health
PCP: Perinatal Care Project
PLA: Participatory Learning and Action
SMS: Short message service
SRQ: Self-Regulation Questionnaire
T2DM: Type 2 diabetes mellitus
TDF: Theoretical Domains Framework
WHO: World Health Organisation

## Footnotes

1 We use the term *intermediate hyperglycaemia* to represent measures of glucose that are categorised as impaired fasting glucose or impaired glucose tolerance according to the WHO definitions outlined in Table 2.
